# Pancreatic Ductal Adenocarcinoma, β-blockers, and Antihistamines: A Clinical Trial Is Needed

**DOI:** 10.1093/function/zqae050

**Published:** 2024-11-15

**Authors:** Jillian G Baker, Erica K Sloan, Kevin D G Pfleger, Peter J McCormick, Cristina Salmerón, Paul A Insel

**Affiliations:** Cell Signalling, School of Life Sciences, C Floor Medical School, Queen’s Medical Centre, University of Nottingham, Nottingham NG7 2UH, UK; Respiratory Medicine, Queen’s Medical Centre, Nottingham University Hospitals, Nottingham NG7 2UH, UK; Drug Discovery Biology Theme, Monash Institute of Pharmaceutical Science, Monash University, Parkville Victoria 3052, Australia; Molecular Endocrinology and Pharmacology, Harry Perkins Institute of Medical Research, Centre for Medical Research, The University of Western Australia, Nedlands, Western Australia 6009, Australia; Department of Pharmacology and Therapeutics, University of Liverpool, Liverpool L69 3GE, UK; Department of Pharmacology, University of California, San Diego, La Jolla, CA 92093, USA; Department of Pharmacology, University of California, San Diego, La Jolla, CA 92093, USA; Department of Medicine, University of California, San Diego, La Jolla, CA 92093, USA

## Introduction—Pancreatic Cancer (Pancreatic Ductal Adenocarcinoma)

**Figure 1.**
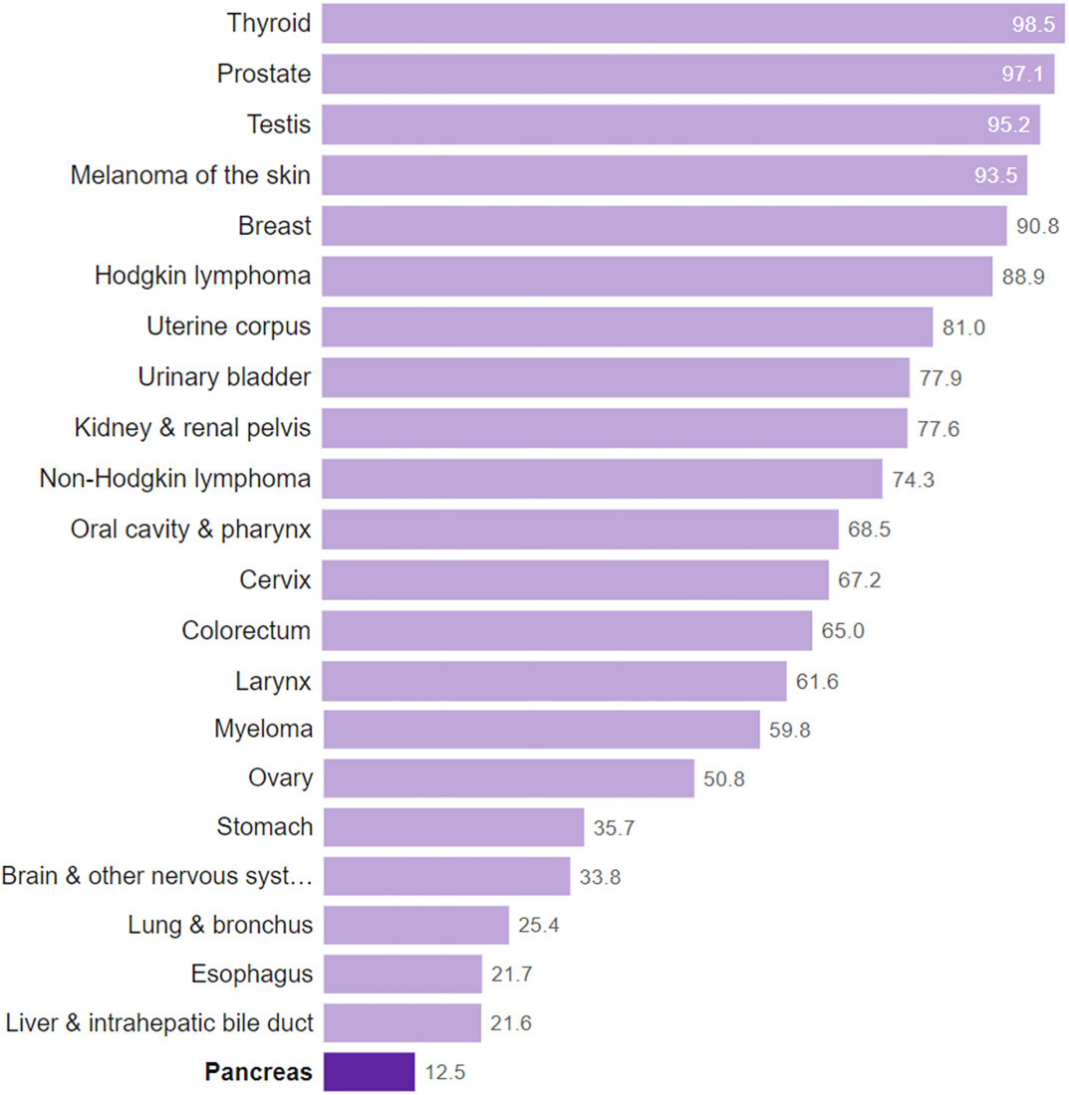
Five-year survival rates in the United States for all cancers listed by the American Cancer Society showing that pancreatic cancer has the lowest 5-year survival of any cancer subtype.

**Figure 2.**
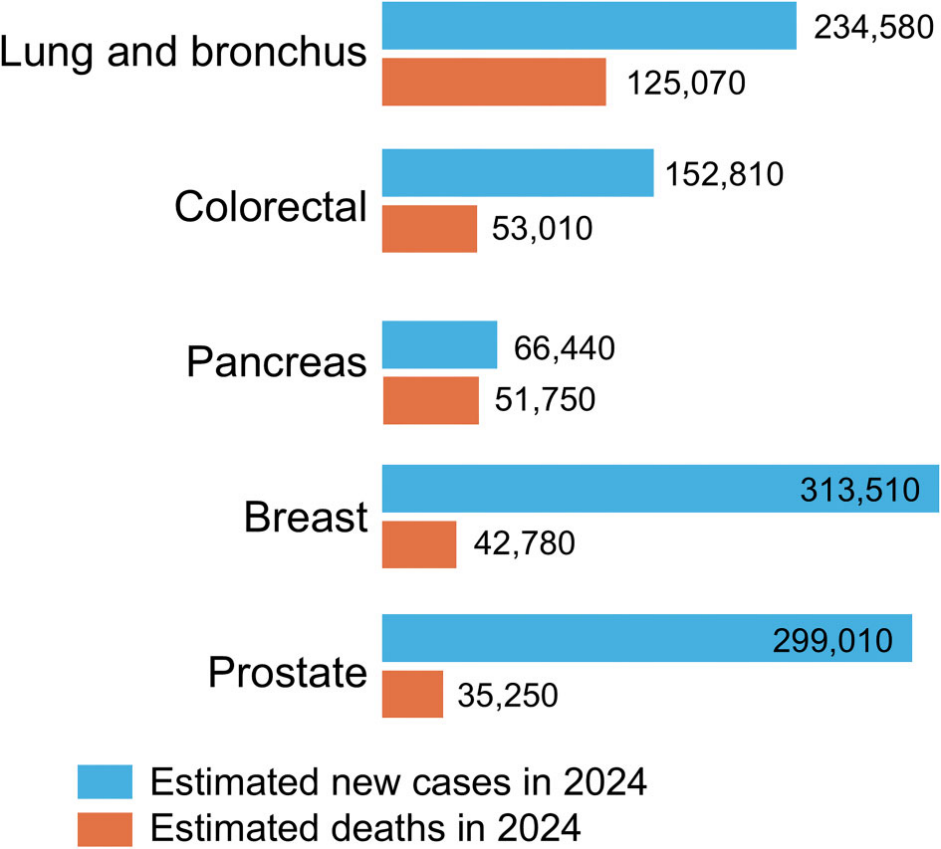
Estimated 2024 incidence (new diagnoses) and mortality rates for the top 5 cancers contributing to cancer death in the United States (from the American Cancer Society https://cancerstatisticscenter.cancer.org, accessed January 19, 2024). These data show that pancreatic cancer is currently the third cause of cancer death in the United States .

Pancreatic ductal adenocarcinoma (PDAC) is one of the most lethal cancers. Globally, each year, almost half a million people die from pancreatic cancer.^1^ PDAC has the lowest 5-year survival of all major cancers (2%-12%^2,3^  https://cancerstatisticscenter.cancer.org, Figure 1) and is predicted to become the second highest cause of cancer deaths in this decade^3,4^ (Figure 2). Approximately 15% of patients with PDAC are diagnosed early and can be treated with surgery and adjuvant chemotherapy; yet their 5-year survival is only 20%-27%.^3^ Most PDAC patients are diagnosed after the cancer has spread beyond the pancreas and have very limited survival: 6-11 months for advanced local PDAC and 2-6 months for metastatic disease.^3^ Overall survival from the time of diagnosis is 5-6 months.^2,3,5^ Therapeutic approaches, such as immunotherapy and targeted therapies, have improved survival of other cancers but have not had a major impact on the survival of PDAC patients.

Current chemotherapeutic regimens (eg, gemcitabine; FOLFIRINOX [folinic acid, fluorouracil, irinotecan, and oxaliplatin]) typically extend life by weeks to months in patients with advanced or metastatic PDAC and are used as adjuvants for those receiving surgery.^3^ However, this treatment is associated with physiological side-effects, a poor quality of life along with the psychological stress of remaining time alive spent in healthcare settings rather than with loved ones. Moreover, there are substantial monetary costs for chemotherapy treatments and healthcare delivered to patients, including to manage side-effects.

Acknowledging the above challenges, we ask: Might drugs approved for other medical conditions be repurposed to aid in the outcome of PDAC patients? We suggest that 2 potential classes of drugs that target G protein-coupled receptors (GPCRs)—β-blockers and H1-antihistamines—may offer such an opportunity. Here, we briefly summarize the evidence supporting this idea and propose the need to test these agents in PDAC patients as a potential means to improve their survival. In our opinion, the body of evidence (from molecular to epidemiological studies) in favor of potential benefit is such that it outweighs the small risks of harm from β-blockers or H1-antihistamines involved in a clinical trial in people with PDAC. We believe that a prospective clinical trial is urgently needed to determine whether these agents can prolong life in those with PDAC, irrespective of the use of other therapies.

## β-Blockers

β-Blockers were developed in the 1960s and are used by millions of patients worldwide. These drugs prolong life in cardiovascular disorders (eg, heart failure, ischemic heart disease) and are first-line agents for the treatment of certain cardiac arrhythmias. β-Blockers are also used in the treatment of hypertension, thyrotoxicosis, portal hypertension, glaucoma, migraine, and anxiety. They are inexpensive generic drugs, have well-recognized side-effects, and are generally well tolerated. β-Blocking actions used for cardiovascular disorders primarily target the cardiac β1-adrenoceptor, but many clinically used drugs are nonselective, binding to both β1- and β2-adrenoceptors and blocking the effects of endogenous hormones/neurotransmitters adrenaline (epinephrine) and noradrenaline (norepinephrine).^6^

In 2008, the nonselective β-blocker propranolol was found to be beneficial for the problematic, predominantly skin vascular tumor and infantile hemangioma and rapidly became the first-line treatment.^7^ β-Blockers can also improve the outcome of cancers (for more detailed reviews, see Hiller et al.^8^ and Carnet et al.^9^). The evidence derives from laboratory studies of tumor cell growth, animal studies that assessed growth of primary tumor size and metastases and epidemiological studies of people prescribed β-blockers for conditions other than cancers.^9^ Unlike laboratory studies in which β-blocker administration is controlled, not all retrospective epidemiology studies evaluate the patients’ exposure to β-blockers (which could range from a single prescription to regular prescriptions and lack measures of adherence, ie, how much of the β-blocker was taken).

Studies in PDAC cells, animal models, and epidemiological analyses (including retrospective analysis of health records in people with pancreatic cancer given β-blockers for other reasons) have shown a benefit from β-blockers.^2,5,10–16^ Complementing such findings, animal studies have shown that increase in catecholamines, either physiologically^10,11^ or by administration,^11,12^ increases growth of cancers, including PDAC.^9^ Several actions of β-blockers may impact on PDAC tumors, including direct effects on tumor cells (by modulating metabolism, proliferation, invasion, and apoptosis), indirectly on the tumor vasculature (eg, angiogenesis) or via impact on anti-cancer immunity. In addition, psychological effects (including stress) can increase endogenous catecholamines and cancer growth, cardiovascular death, and suicide, particularly with poor prognosis cancers, including PDAC.^9–13,16,17^

β2-Adrenoceptors are the most common subtype implicated in most cancer growth and metastasis. Both β1- and β2-adrenoceptors are present in PDAC tumors but human PDAC cell lines express 10-fold higher expression of β2- than β1-adrenoceptor mRNA.^14^ Propranolol, a nonselective β-blocker, has been most often used in studies of a variety of cancers, eg,^11,12^ but carvedilol, another nonselective β-blocker, also has anti-cancer effects.^18^

Key properties for a β-blocker in the treatment of cancer are likely high affinity and long duration of β-blockade (to minimize catecholamine-promoted cancer growth or metastasis). The optimal compound may be a nonselective β-blocker with a long duration of action and without agonist activity. Propranolol has a medium duration of action at the cellular level. Carvedilol has a longer duration of action^19^ and longer plasma half-life but is a weak partial agonist in some model systems; however, this weak agonistic activity may not yield physiological or clinically meaningful effects.^20^

## H1-antihistamines

Evolving evidence suggests that another class of widely used drugs, H1-antihistamines, which are approved for allergic conditions, may be beneficial for cancers, including PDAC. Initially developed in the 1940s, H1-antihistamines are generally safe and are available over the counter worldwide.

The idea that histamine has a role in cancer progression and that H1-antihistamines might be beneficial in treating cancers is not new.^21^ Anti-tumor effects of antihistamines were first noted in the 1970s by phenothiazines that block histamine (eg, promethazine) and antipsychotics (eg, chlorpromazine) in leukemia and melanoma.^21^ Based on in vivo experimental studies and retrospective epidemiological studies, antihistamines were suggested to reduce cancer cell proliferation, migration, invasion, and metastasis and improve survival via effects that alter immune, inflammatory, and vascular activity as well as impact on tumor cells (reviewed in Nguyen et al.^22^). H1-antihistamines may also enhance cancer immunotherapy.^23^

Such anti-cancer activity of H1-antihistamines may not be a general class effect. Actions by cationic amphiphilic antihistamines (eg, ebastine, loratadine and its active metabolite desloratadine) are most effective; some benefits may be histamine-H1 receptor independent.^24,25^ The use of H1-antihistamines has been associated with increased survival in retrospective epidemiological population studies of people given anti-histamines for other reasons, including in ovarian cancer,^26^ lung cancer,^24,27^ multiple cancers,^25^ and reduced incidence of hepatocellular carcinoma.^28^ Some studies report a dose-response effect with increased use of antihistamines having greater benefit.^27,28^ A meta-analysis of several cancer types found that survival in PDAC was improved in those taking H1-antihistamines,^25^ and a matched cohort study showed a reduction in disease progression and increased survival in patients treated with cationic amphiphilic Hi-antihistamines (from 6 to 16 months^29^). Other epidemiological and experimental studies implicate a role for H1-histamine receptors in PDAC biology and benefit of H1-antihistamines in PDAC.^25,30–32^

The choice of an antihistamine for the treatment of cancer should be a long-acting histamine H1 receptor cationic amphiphilic antagonist, preferably second-generation H1-antihistamines, which have less ability to cross the blood brain barrier and produce CNS effects. Desloratadine/loratadine appear to have the best current evidence for treating cancers.

## Is It Time for Trials That Assess Efficacy of β-blockers and H1-antihistamines in PDAC Patients?

Based on the evolving evidence of benefit of β-blockers and H1-antihistamines in PDAC, is it now time for a clinical trial? Given the poor survival rate for PDAC, is it ethical to wait longer to initiate such trials? Repurposing inexpensive, generic drugs is not appealing to pharmaceutical companies so such trials will likely require initiation and funding by governmental entities, and philanthropic or charitable organizations. We believe that improved mortality by such agents could likely be rapidly determined, perhaps within months, and associated with minimal adverse effects. If successful, β-blockers and H1-antihitamines could become standard of care therapy from the time of PDAC diagnosis and tested in combination with other therapies (eg, drugs, radiotherapy, surgery, etc.).

A few trials of β-blockers in PDAC are in progress: (1) treatment with propranolol and chemotherapeutic agents in patients with liver/gallbladder/pancreas cancer (the BLOCKED trial, NCT05451043, now recruiting); (2) treatment with propranolol and the nonsteroidal anti-inflammatory drug etodolac (NCT03838029); (3) treatment with any β-blocker with aspirin, metformin, ACE-inhibitors, and statins (NCT04245644^9^); (4) PROSPER trial (DRKS00014054; EudraCT number: 2018-000415-25), a placebo-controlled phase-2 trial testing propranolol and etodolac, limited to surgically removable PDAC tumors, thus excluding the majority of PDAC patients as most are diagnosed with advanced or metastatic disease^33^; and (5) a trial evaluating the safety of a 10-day course of propranolol in a small preoperative population (NCT06145074) and thus not applicable to most PDAC patients.

Given the poor survival of PDAC patients, trials of β-blockers and H1-antihistamines need not be long. Patients could be randomized at the time of PDAC diagnosis to one of 4 arms: a placebo, a standard dose of β-blocker, H1-antihistamine, or β-blocker + H1-antihistamine, irrespective of other treatments that might be later initiated.^8^ Such a trial would rapidly determine whether either medication had benefit or whether additional benefit occurs in those receiving both (since β-blockers and an antihistamine may affect different aspects of cancer biology: tumor proliferation, migration, invasion, metastasis, and effects that alter immune, inflammatory, and vascular activity). Propranolol (or carvedilol) and desloratadine (or loratadine) are potential drugs for such a trial. Efficacy could be assessed simply by survival, from the time of diagnosis to death, and with a measure of patient's quality of life, would involve minimal efforts by the patients and should not be costly to conduct. Readouts of efficacy (eg, tumor growth, metastases, with, eg, disease markers, scans, etc.) might also be used but would add to the cost of the trial. Given the widespread use and safety of these medications, the only contraindication might be in patients with known allergy to the drugs or with asthma (due to the risk of β-blocker-related bronchospasm and loss of asthma exacerbation rescue), and exclusion of patients already receiving β-blockers or H1-antihistamines. Subsequent studies could identify optimal β-blocker and antihistamine combinations and doses or whether the drugs are more effective in PDAC patients receiving certain other therapies.

How many more people with PDAC will die before we determine whether these widely available and well-tolerated drugs can prolong life for minimal physiological, psychological, and healthcare delivery cost? Given the body of evidence, in our opinion, the potential gain from such a trial far outweighs the risk to patients. Ethically, it seems obvious that the time has come for such a quick, relatively inexpensive, and simple trial.
